# Tolerability and safety of magrolimab (ONO-7913) in Japanese patients with advanced or metastatic solid tumors: a phase 1, open-label, uncontrolled, dose-escalation study

**DOI:** 10.1186/s13104-026-07787-6

**Published:** 2026-03-26

**Authors:** Takafumi Koyama, Toshio Shimizu, Shunsuke Kondo, Yuki Katsuya, Kazuki Sudo, Tatsuya Yoshida, Kan Yonemori, Keiji Matsumoto, Noboru Yamamoto

**Affiliations:** 1 Department of Experimental Therapeutics, National Cancer Center Hospital, 5-1-1 Tsukiji, Chuo-ku, Tokyo, 104-0045 Japan; 2 Department of Medical Oncology, National Cancer Center Hospital, Tokyo, Japan; 3 Department of Thoracic Oncology, National Cancer Center Hospital, Tokyo, Japan; 4 Oncology Clinical Development Planning, ONO Pharmaceutical Co., Ltd., Osaka, Japan; 5 Present Address: Department of New Experimental Therapeutics and International Cancer New Drug Development Center, Kansai Medical University Hospital, Osaka, Japan; 6 Present Address: Department of Medical Oncology, Tokyo Women’s Medical University, Tokyo, Japan

**Keywords:** Advanced or metastatic solid tumors, Anti-CD47 monoclonal antibody, Phase 1 study, Japanese patients, Magrolimab

## Abstract

**Objective:**

Magrolimab (ONO-7913) is an anti-cluster of differentiation 47 (CD47) monoclonal antibody. This was a phase 1, open-label, uncontrolled, dose-escalation study that assessed the tolerability and safety of intravenous magrolimab (priming dose: 1 mg/kg; maintenance dose: 20 mg/kg [Cohort 1] or 30 mg/kg [Cohort 2]; 28-day treatment cycle) in Japanese adult patients with histologically or cytologically confirmed advanced or metastatic solid tumors, ≥ 1 measurable lesion, an Eastern Cooperative Oncology Group performance score of 0–1, and an expected survival ≥ 3 months.

**Results:**

Seven patients were enrolled and received magrolimab (Cohort 1, *n* = 4; Cohort 2, *n* = 3) with the median follow-up of 170.0 (34–491) days. All 7 patients discontinued the magrolimab monotherapy, and 4 (57.1%) patients completed the study as per the protocol. No dose-limiting toxicities were observed in both cohorts, and the maximum tolerated dose was not reached. All patients experienced treatment-emergent adverse events (TEAEs) but not any serious adverse events, TEAEs leading to treatment discontinuation or interruption, or TEAE-related deaths. No complete or partial responses occurred, while the disease control rate was 42.9% (best overall response: stable disease, *n* = 3; disease progression, *n* = 3; not evaluable, *n* = 1). Magrolimab was well tolerated in Japanese patients with advanced or metastatic solid tumors.

*Trial registration*: NCT04403308, submitted on May 21, 2020.

**Supplementary Information:**

The online version contains supplementary material available at 10.1186/s13104-026-07787-6.

## Introduction

Cluster of differentiation 47 (CD47) on cancer cells causes the cancer cells to escape phagocytosis from macrophages by binding to signal regulatory protein alpha (SIRPα) [1–4]. Magrolimab (ONO-7913; formerly, Hu5F9-G4) is an anti-CD47 monoclonal antibody that enhances cancer cell phagocytosis by targeting the CD47-SIRPα system [5]. This further improves antigen presentation to T cells and elicits anti-tumor immune responses [5, 6]. Magrolimab contains a human immunoglobulin G4 isotope inefficient at Fc-dependent recruitment, which decreases the toxicity on healthy CD47-expressing cells [5]. Preclinical studies have reported the safety profile of magrolimab, though a dose-dependent anemia (not associated with intravascular hemolysis and resolved spontaneously) with reticulocytosis and spherocytosis was observed [5].

The promising preclinical results led to clinical trials for magrolimab monotherapy in patients with solid tumors. Specifically, a phase 1, United States (US)-based, dose-escalation study for advanced solid tumors or lymphoma showed the tolerability of magrolimab [7]. The maximum tolerated dose (MTD) for the priming dose was 1 mg/kg, while MTD for the maintenance dose was not reached. However, safety and tolerability of magrolimab in Japanese patients had not been assessed.

Here we present the results of a phase 1, dose-escalation study conducted in Japanese patients to assess the safety and tolerability of magrolimab monotherapy in patients with advanced or metastatic solid tumors.

## Methods

### Study design

This was a phase 1, open-label, uncontrolled, dose-escalation study (ONO-7913-01 study; ClinicalTrials.gov No. NCT04403308). Magrolimab was administered at the priming dose (1 mg/kg) and the maintenance dose (20 mg/kg in Cohort 1 or 30 mg/kg in Cohort 2) until the patient met the discontinuation criteria or completed 24 months of follow-up, whichever occurred first (Supplementary Fig. S1), determined as per the recommendation dose (30 mg/kg) of the previous study [7]. Discontinuation criteria included progressive disease (PD), worsening of symptoms due to disease progression, dose-limiting toxicities (DLTs), or the judgement by the investigator. DLTs were defined as following adverse events (AEs) that occurred during Cycle 1: grade 4 anemia, grade 3 anemia which is caused by hemolysis restricting or interfering with activities of daily living, grade 4 neutrophil count decreased persisting for more than 7 days, grade 4 platelet count decreased or that requiring platelet transfusion, febrile neutropenia with neutrophil count of < 1000/mm^3^ and a single temperature (> 38.3 °C) or a sustained high temperature (≥ 38 °C more than 1 h), and grade > 3 nonhematologic AEs. In addition, Prophylactic administration against infusion-related reactions was performed for the initial 2 doses of magrolimab, including acetaminophen 650 mg and/or diphenhydramine 25–50 mg or equivalent and, if necessary, antihistamine or corticosteroid use.

Following criteria were applied when 3 patients enrolled in Cohort 1 completed a DLT assessment in Cycle 1: if none of 3 experienced a DLT, Cohort 2 is to be initiated; if 1 of 3 experienced a DLT, 3 additional patients is to be enrolled in Cohort 1 and received the DLT assessment; if ≥ 2 of 3 experienced a DLT, the evaluation of Cohort 1 is to be stopped. For each cohort, if 0 of 3 or 1 of 6 patients experienced a DLT, the dose was determined to be tolerated.

### Eligibility criteria

Patients were included if they were ≥ 20 years, had a histologically or cytologically confirmed advanced or metastatic solid tumor, had ≥ 1 measurable lesion on diagnostic imaging within 14 days before the first administration of magrolimab (Response Evaluation Criteria in Solid Tumors [RECIST] guideline v1.1), had an Eastern Cooperative Oncology Group (ECOG) performance score of 0–1, had an expected survival ≥ 3 months at enrollment, were intolerant of or irresponsive to standard therapy or if no standard therapy was available, and had acceptable hematological and biochemistry parameters per pre-defined levels. Patients with multiple cancers, a history of serious allergy, brain metastasis, or other significant medical condition(s) were excluded.

### Endpoints

The primary endpoint was the tolerability of magrolimab. Key secondary endpoints included the safety and pharmacokinetic (PK) profile. Safety assessments included treatment-emergent AEs (TEAEs) and serious AEs (SAEs), defined with the National Cancer Institute Common Terminology Criteria for Adverse Events v5.0, Japanese edition (Japan Clinical Oncology Group), laboratory tests, peripheral blood smear, vital signs, 12-lead electrocardiogram (ECG), and anti-drug antibodies (ADAs). The categories of specific AEs included infusion-related reactions, anemia/extravascular hemolysis, transient RBC agglutination, pneumonia/interstitial lung disease, and anemia/neutropenia/thrombocytopenia. PK endpoints included concentrations and PK parameters of serum magrolimab from day 8 to pre-dose on day 15 of Cycle 1, measured by enzyme-linked immunosorbent assay. Exploratory endpoints of efficacy included overall response rate (ORR), disease control rate (DCR), progression-free survival (PFS), best overall response (BOR), and percent change (and maximum percent change) in sum of tumor diameters of target lesions.

### Statistical analysis

Based on the 3 + 3 cohort design [8], we planned to enroll 3 to 6 patients per cohort with a maximum of 12 patients. Safety, efficacy, and emerging ADAs were examined in the safety population who received at least 1 dose of magrolimab. Frequency distributions and summary statistics were used for baseline characteristics, treatment compliance, and safety endpoints. AEs were coded using the Medical Dictionary for Regulatory Activities (MedDRA) terms. The proportions of patients with ORR, DCR, and BOR were calculated by cohort with 2-sided 95% Clopper-Pearson confidence intervals (CIs).

## Results

### Study population

In total, 7 patients were enrolled (Cohort 1, *n* = 4; Cohort 2, *n* = 3) from July 2020 to February 2021. All 7 discontinued the magrolimab monotherapy due to disease progression. Four (57.1%) patients completed the study as per the protocol (Cohort 1, *n* = 2; Cohort 2, *n* = 2). Two patients in Cohort 1 discontinued the study (loss to follow-up, *n* = 1; death, *n* = 1); 1 patient in Cohort 2 withdrew consent. One patient (Cohort 1) was excluded from the DLT assessment due to early discontinuation (day 7, Cycle 1) prior to the first maintenance dose of 20 mg/kg. The median (minimum–maximum) follow-up period was 170.0 (34–491) days.

Baseline characteristics are shown in Table 1. At the initial diagnosis, 1 patient had Stage IIA disease, 1 patient had Stage IIIB disease, and 5 patients had Stage IV disease. No patients were treatment naive and all patients had an ECOG performance status of 0.

**Table 1:** Baseline characteristics

Characteristic	Cohort 1, *n* = 4	Cohort 2, *n* = 3	Total, *n* = 7
Female sex, n (%)	3 (75.0)	1 (33.3)	4 (57.1)
Age, years			
Mean (SD)	64.0 (7.4)	62.7 (10.0)	63.4 (7.8)
Median (min–max)	67.0 (53–69)	59.0 (55–74)	66.0 (53–74)
BMI, kg/m^2^			
Mean (SD)	19.53 (0.85)	22.51 (3.66)	20.81 (2.71)
Median (min–max)	19.78 (18.3–20.3)	21.67 (19.3–26.5)	19.80 (18.3–26.5)
Tumor type, n (%)			
Colorectal cancer	3 (75.0)	0	3 (42.9)
Thymoma	1 (25.0)	0	1 (14.3)
Intrahepatic cholangiocarcinoma	0	2 (66.7)	2 (28.6)
Thymic carcinoma	0	1 (33.3)	1 (14.3)
Location of primary disease			
Colon	2 (50.0)	0	2 (28.6)
Rectum	1 (25.0)	0	1 (14.3)
Thymus	1 (25.0)	1 (33.3)	2 (28.6)
Intrahepatic bile duct	0	2 (66.7)	2 (28.6)
Duration from initial disease diagnosis to registration, days			
Mean (SD)	61.3 (37.9)	29.0 (13.6)	47.4 (32.8)
Median (min–max)	49.1 (30.6–116.3)	22.3 (20.0–44.6)	44.5 (20.0–116.3)
Stage			
IIA	0	1 (33.3)	1 (14.3)
IIIB	1 (25.0)	0	1 (14.3)
IV	3 (75.0)	2 (66.7)	5 (71.4)
Prior cancer specific surgeries, yes, n (%)	4 (100.0)	1 (33.3)	5 (71.4)
Prior radiotherapy, yes, n (%)	0	2 (66.7)	2 (28.6)
Prior lines of therapy			
1	0	0	0
2	1 (25.0)	0	1 (14.3)
3	0	2 (66.7)	2 (28.6)
≥ 4	3 (75.0)	1 (33.3)	4 (57.1)
Mean (SD)	5.8 (3.3)	3.3 (0.6)	4.7 (2.7)
Median (min–max)	6.0 (2–9)	3.0 (3–4)	4.0 (2–9)
ECOG performance status, n (%)			
0	4 (100.0)	3 (100.0)	7 (100.0)
1	0	0	0
Recent recurrence, yes, n (%)	2 (50.0)	0	2 (28.6)
*Location of recurrence*			
Lung	1 (50.0)	0	1 (50.0)
Lymph node	1 (50.0)	0	1 (50.0)

BMI, body mass index; ECOG, Eastern Cooperative Oncology Group; max, maximum; min, minimum; SD, standard deviation; TNM, tumor, node, metastasis

^a^Data on TNM stage were collected at first diagnosis

### Safety

In the first 3 patients in Cohort 1 (20 mg/kg), DLTs were not observed. Subsequently, patients were assigned to Cohort 2 (30 mg/kg). During the study, no DLTs were observed in both Cohort 1 (*n* = 4) and 2 (*n* = 3); the MTD was not reached.

TEAEs were reported in all 7 patients (Table 2). The TEAEs occurred in ≥ 2 patients were increased blood bilirubin (*n* = 5, 71.4%), anemia (*n* = 5, 71.4%), headache (*n* = 5, 71.4%), nausea (*n* = 3, 42.9%), infusion-related reaction (*n* = 3, 42.9%), vomiting (*n* = 2, 28.6%), pyrexia (*n* = 2, 28.6%), and arthralgia (*n* = 2, 28.6%). The majority of TEAEs were grade 1 or 2 and could be managed with or without intervention. One patient in Cohort 1 had treatment-related grade 3 anemia (spontaneously resolved), and grade 1 extravascular hemolysis 13 days after the onset of anemia. No patients who experienced anemia required blood transfusion. Infusion-related reactions, all grade 1 or 2, occurred in 3 patients (42.9%; Cohort 1, *n* = 1; Cohort 2, *n* = 2) on day 8 of Cycle 1, resolved within 2 days. One patient with grade 2 infusion-related reaction received acetaminophen.

**Table 2:** TEAEs, overall and by cohort

SOC PT	Cohort 1, *n* = 4		Cohort 2, *n* = 3		Total, *n* = 7	
	Any grade, *n* (%)	Grade 3, *n* (%)^a^	Any grade, *n* (%)	Grade 3, *n* (%)^a^	Any grade, *n* (%)	Grade 3, *n* (%)^a^
Any TEAE	4 (100.0)	1 (25.0)	3 (100.0)	0	7 (100.0)	1 (14.3)
Investigations	4 (100.0)	0	3 (100.0)	0	7 (100.0)	0
Blood bilirubin increased	4 (100.0)	0	1 (33.3)	0	5 (71.4)	0
ALT increased	0	0	1 (33.3)	0	1 (14.3)	0
AST increased	0	0	1 (33.3)	0	1 (14.3)	0
Blood bilirubin unconjugated increased	0	0	1 (33.3)	0	1 (14.3)	0
Blood creatinine increased	1 (25.0)	0	0	0	1 (14.3)	0
Lymphocyte count decreased	0	0	1 (33.3)	0	1 (14.3)	0
Platelet count decreased	0	0	1 (33.3)	0	1 (14.3)	0
Blood and lymphatic system disorders	4 (100.0)	1 (25.0)	2 (66.7)	0	6 (85.7)	1 (14.3)
Anemia	4 (100.0)	1 (25.0)	1 (33.3)	0	5 (71.4)	1 (14.3)
Extravascular hemolysis	1 (25.0)	0	0	0	1 (14.3)	0
Red blood cell agglutination	0	0	1 (33.3)	0	1 (14.3)	0
Nervous system disorders	3 (75.0)	0	2 (66.7)	0	5 (71.4)	0
Headache	3 (75.0)	0	2 (66.7)	0	5 (71.4)	0
GI disorders	2 (50.0)	0	2 (66.7)	0	4 (57.1)	0
Nausea	1 (25.0)	0	2 (66.7)	0	3 (42.9)	0
Vomiting	2 (50.0)	0	0	0	2 (28.6)	0
General disorders and administration site condition	1 (25.0)	0	2 (66.7)	0	3 (42.9)	0
Pyrexia	0	0	2 (66.7)	0	2 (28.6)	0
Fatigue	1 (25.0)	0	0	0	1 (14.3)	0
Injury, poisoning, and procedural complications	1 (25.0)	0	2 (66.7)	0	3 (42.9)	0
Infusion-related reaction	1 (25.0)	0	2 (66.7)	0	3 (42.9)	0
Musculoskeletal and connective tissue disorders	1 (25.0)	0	2 (66.7)	0	3 (42.9)	0
Arthralgia	0	0	2 (66.7)	0	2 (28.6)	0
Back pain	1 (25.0)	0	0	0	1 (14.3)	0
Infections and infestations	2 (50.0)	0	0	0	2 (28.6)	0
Cystitis	1 (25.0)	0	0	0	1 (14.3)	0
Urinary tract infection	1 (25.0)	0	0	0	1 (14.3)	0
Metabolism and nutrition disorders	2 (50.0)	0	0	0	2 (28.6)	0
Decreased appetite	1 (25.0)	0	0	0	1 (14.3)	0
Dehydration	1 (25.0)	0	0	0	1 (14.3)	0
Skin and subcutaneous tissue disorders	1 (25.0)	0	1 (33.3)	0	2 (28.6)	0
Pruritus	0	0	1 (33.3)	0	1 (14.3)	0
Rash	1 (25.0)	0	0	0	1 (14.3)	0
Skin disorder	0	0	1 (33.3)	0	1 (14.3)	0

TEAEs were included up to 30 days after the start of study treatment or the start of post-treatment, whichever was earlier

ALT, alanine aminotransferase; AST, aspartate aminotransferase; GI, gastrointestinal; PT, preferred term; SOC, system organ class; TEAE, treatment-emergent adverse event

^a^No patient experienced adverse events of Grade 4 or 5

Specific AEs were reported in 6 (85.7%) patients, excluding 1 patient in Cohort 2. These were anemia (*n* = 5, 71.4%; onset, ≤ 2 months), blood bilirubin increased (*n* = 5, 71.4%), blood bilirubin unconjugated increased (*n* = 1, 14.3%), extravascular hemolysis (*n* = 1, 14.3%) each, infusion-related reaction (*n* = 3, 42.9%), and rash (*n* = 1, 14.3%). All events were grade 1 or 2, except for grade 3 anemia in 1 patient in Cohort 1. No events of grade 4 or 5 were observed. All reported specific AEs were considered related to magrolimab.

No patients had experienced SAEs or TEAEs leading to treatment discontinuation, treatment interruption, or dose reduction. All AEs occurred by day 28, except in 1 patient each in Cohort 2 for pyrexia and RBC agglutination, both of which occurred between days 29 and 56 and in 1 patient in Cohort 2 with arthralgia which occurred day 57 onwards. Three patients (42.9%) died during the study due to disease progression.

Among the time courses of laboratory values, decrease in RBC count, hemoglobin, hematocrit, and haptoglobin and increase in reticulocytes, indirect bilirubin, total bilirubin, and D-dimer were observed after the start of magrolimab treatment, with no notable changes in any other laboratory value. No patients had an alanine aminotransferase (ALT) or aspartate aminotransferase (AST) level > 3 times the upper limit of normal (ULN) or a total bilirubin level > 2 times ULN after the start of treatment. Based on evaluation of peripheral blood smears, 10–19% (1+) RBC agglutination was observed in 5 patients after the start of treatment, but ≥ 20% (2+) RBC agglutination did not occur. Spherocytes, RBC fragments/schistocytes, nucleated RBCs, abnormal RBCs, and/or platelet aggregation were not observed in any patient.

There were no clinically meaningful changes from baseline in vital signs and ECG after the start of magrolimab treatment.

One patient in Cohort 2 had treatment-induced ADA; converted to ADA positive on day 1 of Cycle 2 and remained ADA positive at the end of the treatment period.

### Pharmacokinetics

**Fig. 1:**
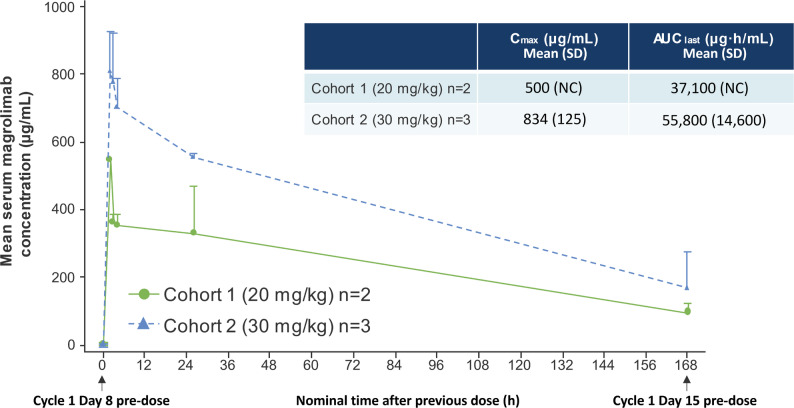
Serum magrolimab concentration-time profile: pre-dose day 8 to pre-dose day 15 of Cycle 1. AUC_last_, area under the concentration-time curve from time 0 to last measurable concentration; C_max_, maximum serum concentration; h, hours; NC, not calculated; SD, standard deviation

Trough serum magrolimab concentrations increased with increasing dose, reaching a maximum at approximately 2 h after initiating magrolimab administration and decreasing overtime (Fig. 1 and Supplementary Table S1). Magrolimab exposure (area under the concentration-time curve from time 0 to last measurable concentration [AUC_last_] and maximum serum concentration [C_max_]) increased in a dose-proportional manner.

### Efficacy

No complete or partial responses (CRs or PRs) were observed; therefore, the ORR was 0%. The BOR was stable disease (SD) in 3 patients (Cohort 1, *n* = 1; Cohort 2, *n* = 2), PD in 3 patients (Cohort 1, *n* = 2; Cohort 2, *n* = 1), and not evaluable (NE) in 1 patient of Cohort 1. The DCR was 42.9% (95% CI: 9.9–81.6%). One patient with colorectal cancer experienced prolonged SD with a PFS of 392 days. The percent change from baseline and maximum percent change from baseline in the sum of tumor diameters of target lesions are shown in Supplementary Fig. S2.

## Discussion

In this study, we evaluated tolerability and safety of magrolimab in Japanese patients with advanced or metastatic solid tumors with limited treatment options. Magrolimab was well tolerated at a priming dose of 1 mg/kg followed by maintenance doses up to 30 mg/kg, with no DLTs. No SAEs or AEs leading to treatment discontinuation occurred. The serum magrolimab concentration and magrolimab exposure (AUC_last_ and C_max_) showed a dose-proportional increase. Time to maximum serum concentration was approximately 2 h.

Frequent TEAEs in this study were similar to the previous phase 1 US-based trial [7]. Among TEAEs, anemia is an on-target AE occurred with blockage of CD47 including magrolimab [9–11]. To mitigate anemia, we primed dose of 1 mg/kg magrolimab to spare older RBCs with younger ones. Expectedly, blood bilirubin occurred in 5 of 7 patients probably due to manifestation of extravascular hemolysis in this study; these events were all grade 1 or 2 and managed with no major safety concerns. Regarding anemia, although 5 of 7 patients experienced anemia of any grade, only 1 patient experienced grade 3 anemia that was resolving spontaneously. Overall, safety profiles of magrolimab treatment were manageable and comparable to the previous US-based trial.

The situation of magrolimab therapy is different between hematologic cancers and solid cancers. In the phase 3 ENHANCE-3 trial, magrolimab therapy plus azacitidine and venetoclax increased a risk of death with grade 5 infections and respiratory events for acute myeloid lymphoma [12]. Due to this futility, development of magrolimab has been discontinued for hematologic cancers. On the other hand, for solid tumors, combination therapy of magrolimab did not result in grade 5 TRAEs [13–16]. Consistently, in this study, magrolimab monotherapy did not induce grade 4 or 5 AEs either. The frequency of toxicity with magrolimab may vary depending on the type of tumor.

In this study, no CRs or PRs were observed, although SD was observed in 1 of 7 patients with a PFS of 392 days. Previous phase 1 trials showed that PRs were observed in 2 of 62 patients for magrolimab or in 1 of 27 patients for AO-176 (another anti-CD47 antibody) [7, 17]. Since the sample size of this study was insufficient compared with those of previous studies, further investigation with large sample size would be required. In addition, considering that magrolimab only suppresses the “don’t eat me” signal, combination therapy with other drugs may be necessary to exert tumor response of magrolimab. Accordingly, some magrolimab combination therapies have been evaluated in clinical trials. For solid cancers, several trials have suggested promising efficacy of the combination therapy such as dotaxel, cetuximab, and nivolumab [13–16]. Combination therapy of magrolimab with chemotherapy and/or immunotherapy might be important, in which magrolimab would strengthen phagocytosis and anti-tumor immune responses.

### Limitations

As this study aimed to assess tolerability and safety in Japanese patients, the number of enrolled patients was insufficient for the statistical analysis.

## Conclusion

Magrolimab was well tolerated at a priming dose of 1 mg/kg followed by a weekly maintenance dose up to 30 mg/kg in Japanese patients with advanced or metastatic solid tumors. Safety profiles were manageable and comparable to the previous phase 1 US-based trial.

## Supplementary Information

Below is the link to the electronic supplementary material.

Supplementary Material 1.

## Data Availability

Any researcher may request ONO Pharmaceutical Co., Ltd. to disclose individual patient level data from clinical studies through Vivli ( [https://vivli.org/](https:/vivli.org) ). Interested researchers should consult [https://www.ono-pharma.com/en/company/policies/clinical\_trial\_data\_transparency\_policy.html](https:/www.ono-pharma.com/en/company/policies/clinical_trial_data_transparency_policy.html) for more information on ONO Pharmaceutical Co., Ltd.‘s Global Policy about the Disclosure of Clinical Study Data.

## Abbreviations

ADA: Anti-drug antibodies
AE: Adverse events
ALT: Alanine aminotransferase
AST: Aspartate aminotransferase
BOR: Best overall response
CD47: Cluster of differentiation 47
CI: Confidence intervals
DCR: Disease control rate
DLT: Dose-limiting toxicities
ECG: Electrocardiogram
ECOG: Eastern Cooperative Oncology Group
MedDRA: Medical Dictionary for Regulatory Activities
MTD: Maximum tolerated dose
ORR: Overall response rate
OS: Overall survival
PFS: Progression-free survival
PK: Pharmacokinetic
PR: Partial response
RECIST: Response Evaluation Criteria in Solid Tumors (guideline v1.1)
SAE: Serious adverse events
SIRPα: Signal regulatory protein alpha
TEAE: Treatment-emergent adverse events
ULN: Upper limit of normal
US: United States
