# Supplementary material for: Tolerability and safety of magrolimab (ONO-7913) in Japanese patients with advanced or metastatic solid tumors: a phase 1, open-label, uncontrolled, dose-escalation study
Source: BMC Res Notes. 2026 Mar 26;19:203. doi: 10.1186/s13104-026-07787-6 (PMC13141484; doi:10.1186/s13104-026-07787-6)
Supplement: Supplementary file 1 — Supplementary Material 1. [file 13104_2026_7787_MOESM1_ESM.doc]

**Supplementary material**

**Supplementary Table S1.** PK Summary statistics: pre-dose on Day 8 to pre-dose on Day 15 of Cycle 1

|  | **Cohort 1** | **Cohort 2** |
| --- | --- | --- |
| C_max_, µg/mL | | |
| n | 2 | 3 |
| Mean (SD) | 550 (NC) | 834 (125) |
| Median (min–max) | 550 (540–560) | 880 (693–930) |
| %CV | NC | 15.0 |
| GeoMean | 550 | 828 |
| %GeoCV | NC | 15.7 |
| T_max_, hours | | |
| n | 2 | 3 |
| Mean (SD) | 2.01 (NC) | 2.33 (0.35) |
| Median (min–max) | 2.01 (1.98–2.03) | 2.18 (2.07–2.73) |
| %CV | NC | 15.2 |
| GeoMean | 2.00 | 2.31 |
| %GeoCV | NC | 14.8 |
| AUC_last_, µg.h/mL | | |
| n | 2 | 3 |
| Mean (SD) | 37,100 (NC) | 55,800 (14,600) |
| Median (min–max) | 37,100 (26,400–47,900) | 56,000 (41,100–70,300) |
| %CV | NC | 26.1 |
| GeoMean | 35,500 | 54,500 |
| %GeoCV | NC | 27.4 |
| T_1/2_, hours | | |
| n | 2 | 3 |
| Mean (SD) | 88.9 (NC) | 77.6 (37.7) |
| Median (min–max) | 88.9 (85.3–92.5) | 92.4 (34.8–106) |
| %CV | NC | 48.5 |
| GeoMean | 88.8 | 69.7 |
| %GeoCV | NC | 66.7 |

AUC_last_, area under the concentration-time curve from time 0 to last measurable concentration; C_max_, maximum serum concentration; CV, coefficient of variation; Geo, geometric; max, maximum; min, minimum; NC, not calculated; PK, pharmacokinetic; SD, standard deviation; T_1/2_, elimination half-life; T_max_, time to maximum serum concentration

**Supplementary Fig. S1** Study design


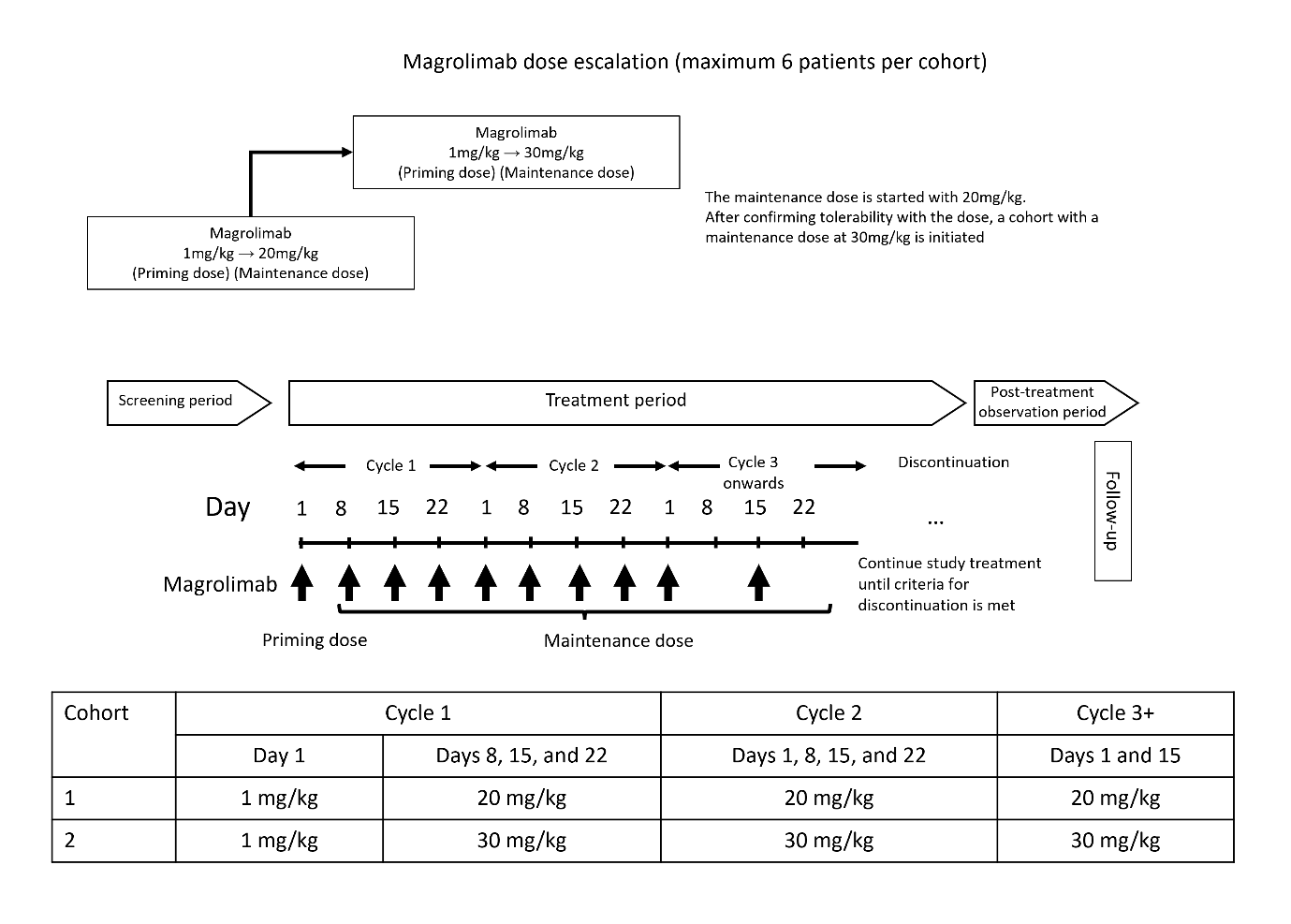


**Supplementary** **Fig. S2 Efficacy results**


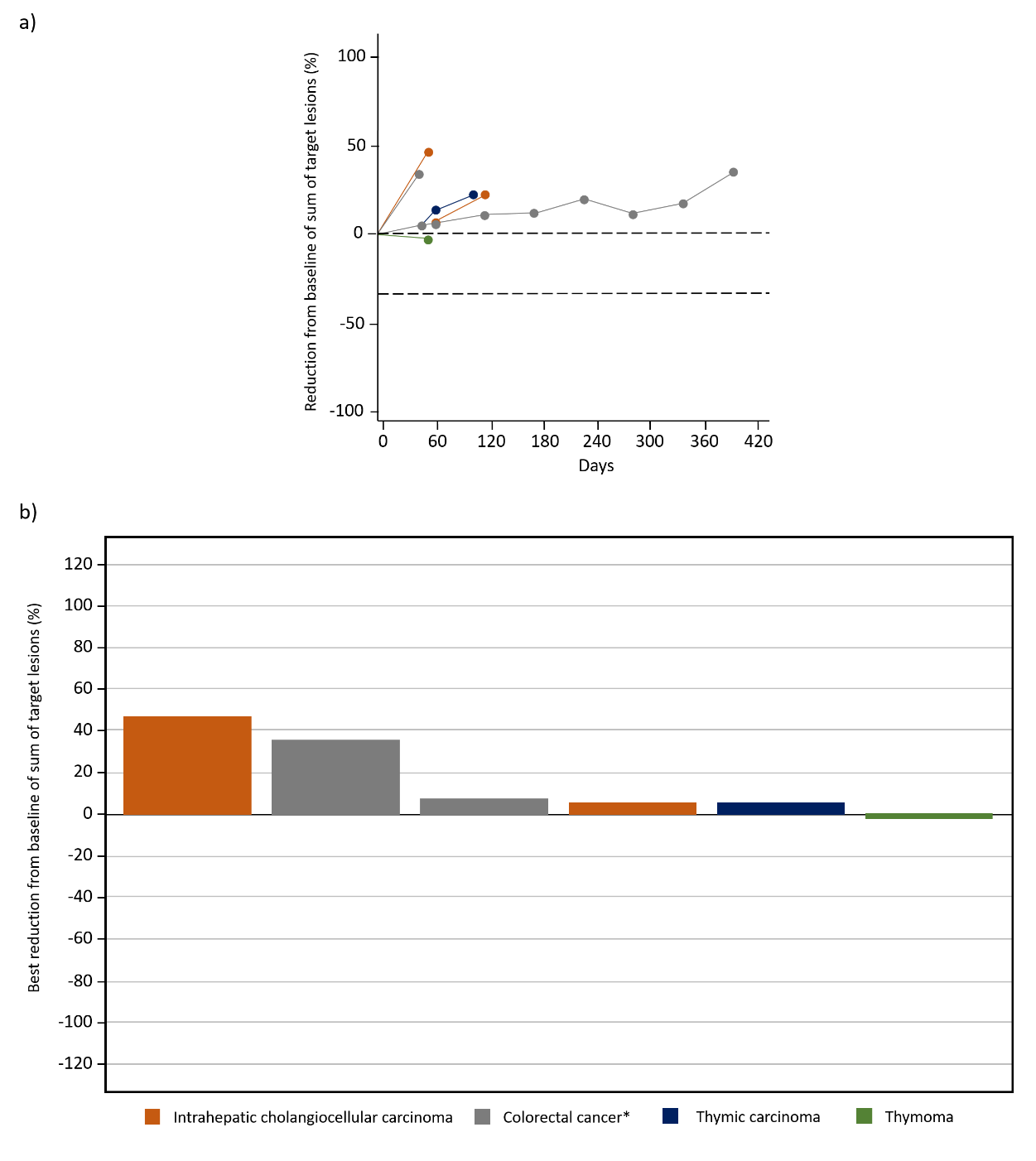


a) Time course of percent change from baseline in the sum of tumor diameters of target lesions and b) Waterfall plot of maximum percent change from baseline in the sum of tumor diameters of target lesions

*One patient with colorectal cancer had no assessment after screening
